# Testing for carryover effects after cessation of treatments: a design approach

**DOI:** 10.1186/s12874-016-0191-6

**Published:** 2016-08-02

**Authors:** S. Gwynn Sturdevant, Thomas Lumley

**Affiliations:** The Department of Statistics, The University of Auckland, Private Bag 92019, Auckland, 1142 New Zealand

**Keywords:** Prehypertension, Cumulative incidence, Survival analysis, Carryover, Randomized trials, Prevention

## Abstract

**Background:**

Recently, trials addressing noisy measurements with diagnosis occurring by exceeding thresholds (such as diabetes and hypertension) have been published which attempt to measure carryover - the impact that treatment has on an outcome after cessation. The design of these trials has been criticised and simulations have been conducted which suggest that the parallel-designs used are not adequate to test this hypothesis; two solutions are that either a differing parallel-design or a cross-over design could allow for diagnosis of carryover.

**Methods:**

We undertook a systematic simulation study to determine the ability of a cross-over or a parallel-group trial design to detect carryover effects on incident hypertension in a population with prehypertension. We simulated blood pressure and focused on varying criteria to diagnose systolic hypertension.

**Results:**

Using the difference in cumulative incidence hypertension to analyse parallel-group or cross-over trials resulted in none of the designs having acceptable Type I error rate. Under the null hypothesis of no carryover the difference is well above the nominal 5 % error rate.

**Conclusions:**

When a treatment is effective during the intervention period, reliable testing for a carryover effect is difficult. Neither parallel-group nor cross-over designs using the difference in cumulative incidence appear to be a feasible approach. Future trials should ensure their design and analysis is validated by simulation.

**Electronic supplementary material:**

The online version of this article (doi:10.1186/s12874-016-0191-6) contains supplementary material, which is available to authorized users.

## Background

Hypertension and diabetes are responsible for significant mortality, morbidity, and cost in both developed and developing countries [1]. Rather than intervening after these high-risk conditions develop, it would be preferable to intervene to prevent incident hypertension and diabetes. In this paper we discuss trial design for evaluating interventions that prevent hypertension or diabetes.

Reliably estimating carryover effects requires that incident hypertension is diagnosed without differential bias by treatment group, and rapidly enough to distinguish zero, short, and long-term carryover. Stephen Senn defines “direct effects” as the effect of a treatment while administered and “residual effects” as forms of carryover which occur after treatment has ceased [2]. We define carryover as these “residual effects.” This paper is a systematic simulation study where we attempt to find more robust methods with which to test a carryover hypothesis by focussing on altering various parameters and analysing Type I error rates. For concreteness, we describe the study designs in terms of systolic blood pressure (BP) and hypertension, but the results transfer to other incident events defined by thresholds in similar ways.

This paper is a systematic simulation study where we attempt to find more robust methods with which to test a carryover hypothesis by focussing on altering various parameters and analysing Type I error rates. For concreteness, we describe the study designs in terms of systolic BP and hypertension, but the results transfer to other incident events defined by thresholds in similar ways.

The question of carryover effects is obviously of interest for intensive, short-duration lifestyle interventions, but the statistical issues were in fact first studied in the context of a pharmacologic intervention. The Trial of Preventing Hypertension (TROPHY) [3] was conducted to determine if two years of treatment with candesartan in prehypertensives reduced the incidence of hypertension over the two years after treatment ceased.

The TROPHY [3] investigators designed a 4 year trial in which 809 subjects with systolic BP measurements between 130 – 139 mm Hg or diastolic BP 85 – 89 mm Hg were randomised to either treatment or placebo for 2 years. BP measurements were taken every 3 months, and diagnosis of hypertension occured when any 3 systolic or diastolic measurements were above the threshold of 140/90 mm Hg. Cumulative incidence in the placebo arm was 63.0 %, and in the treatment arm 53.2 %. The investigators concluded that “the effect of active treatment on delaying the onset of hypertension can extend up to 2 years after the discontinuation of treatment”.

### Issues in the TROPHY design

Due to random variation, the control arm of the trial is more likely to have measurements above the threshold than the treatment group for the initial 2 years. As TROPHY diagnosed hypertension when 3 measurements were above the threshold, which is an unusual definition of hypertension, it resulted in a systemic bias in the design. This bias prior to the onset of the carryover period resulted in more diagnoses in the control arm of the study. The control arm is partially diagnosed after treatment ends. Participants removed from the control arm during treatment makes the subsequent two years not comparable. Simulations suggest that TROPHY had an 80 % Type I error rate [4]. Meltzer (2006) also criticised TROPHY’s design as having an “idiosyncratic primary endpoint [that] seriously impairs external applicability.” Persell and Baker (2006) noted that cumulative diagnosis rates would differ even with identical underlying BP.

### Possible solutions

The Prostate Cancer Prevention Trial is a parallel-group trial that had to account for many sources of bias [5]. In this trial 18,882 men were randomised to treatment with finasteride or placebo. Unfortunately, finasteride impacts upon PSA levels that are used to diagnose prostate cancer. With this complication, it was likely that there would be differences in the rates of biopsies in the treatment and control arms of the study. To address this issue, the investigators used two techniques. First, they required all participants undergo biopsies at the end of the 7 year study. With this adjustment, the only remaining concern was having similar numbers of biopsies for the duration of the study, prior to the end. The solution the investigators proposed was to have differing thresholds used for PSA levels between the treatment and control arms of the study. High PSA levels were sent to the statistical centre to determine if a biopsy was necessary [5].

The TROPHY trial investigators could have used a similar technique to address differing levels of diagnosis rates. Although impractical, ambulatory BP (were participants wear a cuff which measures their BP every 15 - 30 minutes over an extended period of time) could be used to accurately determine rates of hypertension at the end of the study [6]. Throughout the study, TROPHY investigators could have utilised differing thresholds for the treatment and control arms of the study determined by a statistical centre to ensure similar diagnosis rates. Unfortunately, the investigators did not recognise the inherent bias, so never considered this solution.

A crossover trial is another possible solution that we considered. It is plausible that this design allows for detecting the existence of carryover, and estimating its cumulative magnitude. The treatment and control arms of the study experience equal times with lowered BP when there is no carryover and the numbers of people diagnosed in both arms would be approximately equal at the same time from the start of treatment. Any carryover effect would result in lower cumulative incidence in the treatment-first arm, by an amount that depends on the magnitude and duration of the carryover effect. Although it is unusual to use a cross-over design for studying an irreversible outcome, other recent examples exist [7, 8].

Simple comparisons of diagnosis rates in crossover trials are frequently used to determine treatment effects [9]. We explore this methodology to see if it correctly identifies carryover. We test an analysis for determining carryover when the effect of treatment is known. This differs from the norm, which determines the effect of treatment in the presence of carryover. We want to determine if detection of carryover is possible solely using design.

Carryover has been an important topic in the literature on cross-over trials, but primarily as a nuisance factor. That is, trials have been designed so that the carryover effect need not be known when estimating the effect of an intervention during the treatment period.

Even with this goal, it is controversial whether existing cross-over designs can usefully handle carryover effects. Stephen Senn cautions that “including carry-over in the model has a disastrous effect on efficiency” [8]. Two-period cross-over designs have been criticised as having low power [10–13]. Tests for carryover involving more complex designs are faulted for unrealistic assumptions [10, 11] — namely, that carryover occurs for at most one period, or that carryover has equal effect throughout all subsequent periods. Stephen Senn laments “that little has appeared... on the subject of modelling for carry-over [sic] that is more grounded in clinical and pharmocological reality” [2].

### Difficulties in measuring carryover

BP varies throughout the day, over the year, and with a range of outside influences and non-negligible measurement error [14–19]. Diagnosing hypertension based on a single measurement will introduce unacceptable levels of noise, but averaging multiple measurements taken over a long interval makes it impossible to localise incident hypertension accurately in time. A practical study design must compromise and define hypertension in terms of a small number of measurements taken at relatively infrequent intervals. A further complication is that, for ethical reasons, an individual who crosses the diagnostic threshold for hypertension must receive open-label treatment that will change all future BP measurements and make their future data effectively useless in diagnosing hypertension.

In this paper we consider a univariate measurement, systolic BP, rather than the bivariate measurement (systolic, diastolic) used in TROPHY and in the prior simulation studies.

In section ‘Methods’ we explain how we generated data, designed studies, and diagnosed hypertension. Section ‘Results and discussion’ discusses pertinent results and section ‘Crossover’ discusses where crossover designs encounter problems. Section ‘Conclusions’ summarises our results and suggests further research.

## Methods

### Generating data

We model systolic BP (*Y*_*it*_ for individual *i* at time *t*) as normally distributed around an individual-specific long-term average trend

1 $$  Y_{it}= a_{i} +b_{i}t + c_{i} X_{it} + d_{i} Z_{it} + \epsilon_{it}  $$

where *Y*_*it*_ is the BP measurement, *a*_*i*_∼*Unif*(125,140), *b*_*i*_∼*N*(0,*Σ*), *c*_*i*_ estimates the treatment effects, *d*_*i*_ estimates the carryover, *X*_*it*_ is 1 if person *i* is on treatment at time t and 0 otherwise, and *Z*_*it*_ starts at 1 when someone stops treatment and decreases linearly to 0 over the carryover period. The reason for the uniform distribution on the individual random intercepts is that entry into the study is based on BP thresholds.

**Fig. 1:**
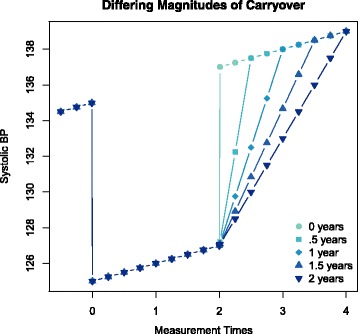
Systolic BP simulation with and without carryover. There are 5 different lengths of carryover — 0, 0.5, 1, 1.5, and 2 years. A carryover of 0 (*blue green*) is a scenario with no carryover effect

Figure 1 shows some possible models for lengths of carryover in a four year study of hypertension. As we can see from the graph, BP is lowered during treatment with medication. At the end of treatment BP returns either quickly to a normal trend, or more gradually, depending upon if carryover does exist and the length. Reliably estimating carryover effects requires that incident hypertension is diagnosed without differential bias by treatment group, and rapidly enough to distinguish zero, short, and long-term carryover. In terms of our model the *Z*_*it*_ are altered.

### Study design

Parallel-group designs have not adequately accounted for the complexities of testing a carryover hypothesis; we try to determine if they can. We completed two sets of simulations — one involving altering inclusion criteria, the other, altering other parameters — then analysis of false positives and power were done in each to determine the effectiveness of the design.

**Fig. 2:**
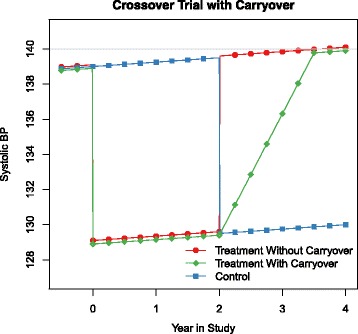
Above, two groups receive treatment first with no carryover and a carryover of 1.5 years, the other group treatment second. Similar diagnosis rates between the treatment first without carryover and treatment second would allow for detection of carryover

In a two-period two-treatment crossover trial half the subjects receive treatment A first then crossover to treatment B [20] as demonstrated by Fig. 2. For our purposes, treatment A lowers BP while treatment B involves placebo only. Here we see 3 possibilities: treatment second, treatment first and 1.5 years carryover, and treatment first and no carryover.

We conducted both parallel-group and crossover simulations to evaluate the tests for the presence of carryover effect and the estimates of its magnitude. Both sets of simulations began with a random number distributed uniformly between 125 – 140 mm Hg as the participants in the TROPHY trial were prehypertensive [3]. From here a trend is used as BP increases over time, [21, 22] we used a trend of 0, 1, and 2 mm Hg per year which is similar to what has been used in simulations which have replicated TROPHY [4, 23]. BP is variable due to both measurement error and intra-individual variability which we combined to assume normally distributed standard deviations of 3, 5, and 7 mm Hg [4, 23]. We used treatment effects of −5 and −10 mm Hg. Measurements were taken either 3 monthly, 6 monthly, or yearly and carryovers of length 0, 0.5, 1, 1.5, and 2 years were assumed. Treatment length was 2 years for the cross-over trial which is the length of time participants received treatment in TROPHY [3]. For the parrallel-group trial, the duration of the treatment was either 1, 1.5, 2, 2.5, or 3 years [4].

Simulations for the parallel-group design also looked at varying the inclusion criteria, by sampling from a uniform distribution with the baseline BP from 110–140, 120–140, or 130–140 mm Hg. We fixed the design at 2 years of treatments [3], 1 mm Hg per year trend in BP [4], and a standard deviation of 5 mm Hg [4]. The carryover duration was 0,.5, 1, 1.5, or 2 years [3], measurements 3 monthly, 6 monthly, or yearly [4].

The simulations produced an estimate of the cumulative incidence of diagnosis in the two trial arms. The Type I error rate (and the power, not reported) were computed from these two cumulative incidences and the sample size using standard formulas for power calculation [24].

A three arm trial with combination parallel and crossover was also simulated where one arm received treatment first, another treatment second, and the third no treatment. The same values and parameters were used for this simulation as those above.

### Rules

In addition to the formula for BP it was necessary to develop criteria to establish when a person became hypertensive. We analysed five feasible criteria for diagnosing hypertension using a threshold of 140 mm Hg: if one measurement was above, if two consecutive measurements were above, if the average of two consecutive measurements were above, if any three measurements were above, and if the average of three consecutive measurements were above. To illustrate the importance of measurement error we also considered a rule that diagnosed hypertension when both the measured systolic pressure and the underlying long-term BP were above threshold. This rule is not of practical use, although it could be implemented by averaging a large number of measurements over a period of days for anyone who had a single measurement over 140 mm Hg.

## Results and discussion

### Parallel-group design

**Fig. 3:**
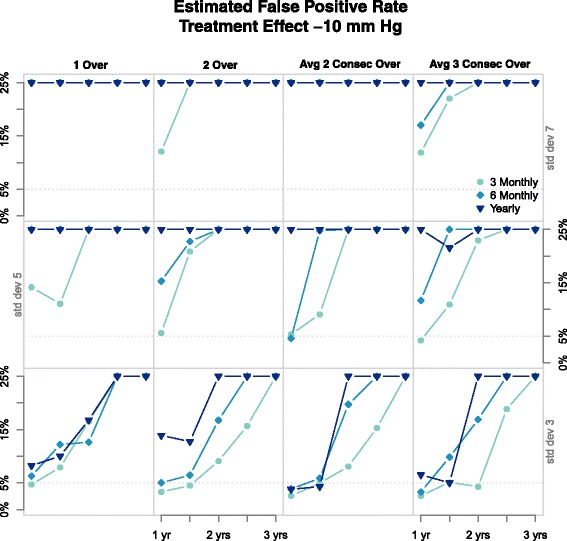
This graph shows estimated rates of false positives in a parallel-group design for the rules mentioned, most are far from the normal 5 %

Figure 3 shows the rates of false positives across four rules studied, which are significantly higher than the accepted rate of 5 *%*. The *x* axis of each graph tells us the length of time participants received treatment, with differing measurement standard deviations found in rows and columns signifying varying rules. The line types distinguish the frequency of measurements, as indicated in the key. All the results have trend of 1 mm Hg per year. Figure 3 shows some general trends: as the error increases the rate of false positives also increases, less frequent measurements result in increased rates of false positives, and as the duration of treatment increases so does the rate of false positives. The last suggests that these designs are unable to distinguish between the treatment period and carryover period. In general, type I error rate is inflated except for the smallest measurement error and shortest period of active treatment. Generalising to a bivariate measurement will make the design perform worse.

Two rules are omitted from the graph. One, where people are diagnosed when 3 measurements are above the threshold, has been studied previously [23, 25, 26], the other is the infeasible rule that uses the true long-term-average BP. This rule is the only one that does achieve close to nominal Type I error rate. Additional files 1 and 2 are the R code used in our simulation process.

### Parallel-design — inclusion criteria

**Fig. 4:**
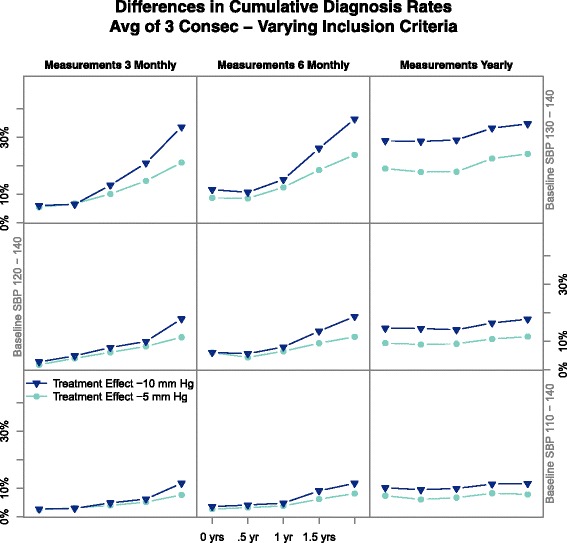
Impact of altering inclusion criteria upon differences in cumulative diagnosis rates in a parallel-group design. The *x* axis indicates the length of carryover

Measurement error can lead to false positive diagnosis only when true BP is relatively close to the threshold, so varying inclusion criteria for baseline long-term-average BP were considered. Figure 4 shows difference in cumulative incidence of diagnosis for three inclusion thresholds (110, 120, 130 mm Hg) in the presence and absence of carryover. Each line colour shows differing treatment effects, as indicated by the key. The 3 columns indicate differing measurement schedules per the labels above, and the *x* axis indicates the length of carryover. Figure 4 demonstrates several results: treatment effects of −10 mm Hg results in fewer people being diagnosed in the treatment arm, lowering the lower bound of the inclusion criteria for baseline systolic BP results in fewer people diagnosed, and more frequent measurements allows limited distinction between short and longer carryovers. Including participants with lower BP reduces the estimated carryover effect under the null hypothesis, but also under the alternative hypothesis. Additional file 3 is the R code used to simulate these trials.

### Crossover design

**Fig. 5:**
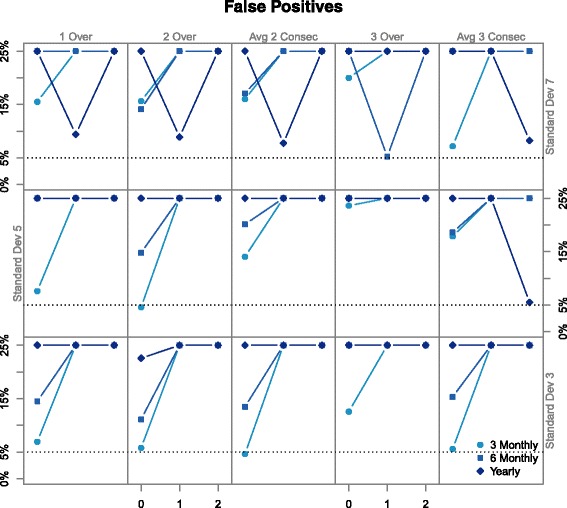
Above are the Type I error rates for differing rules and standard deviations in a crossover design. The *x* axis denotes trend and differing line types indicate measurement schedules

Figure 5 shows the Type I error rate for five rules sudied, all of which are higher than the nominal 5 % rate. The *x* axis of each graph denotes the trend in BP, differing line types indicate measurement schedules, per the key. Each rule recieves its own column, while standard deviations are in rows. As none of the rules even has suitable Type I error rate, we do not present results on the power of the tests or the estimates of carryover magnitude. The rule which involves removing variation is not included; even this design had high Type I error. Additional files 4 and 5 are the R code used to simulate crossover trials.

Figure 5 has a few notable trends. In most graphs, the trials represented by points with trend 0 mm Hg per year (those to the left above 0 on the *x* axis) have false positive rates close to the boundary of 5 %. In most graphs, of those with trend 0 mm Hg per year, the lighter points are closer to 5 % which shows that more frequent measurements are more likely to have appropriate rates of false positives. Trials on the top row (simulated with an error of 7 mm Hg) results in diagnosis rates with little consistency.

### Combination of parallel and crossover

As the parallel group design has positive bias [4, 23] and the crossover design has negative bias (which will be discussed in section ‘Crossover’), there is the potential for combining the two and having the bias cancel out. The combination design would be a two period, three arm crossover trial in which 1 arm receives treatment for the first period and placebo the second, another receives placebo for both periods, and the last placebo followed by treatment.

Our simulations showed that this combination cannot be made to work reliably. The rules tested demonstrated inconsistent differences in relation to increasing amounts of carryover (data not shown). Additional files 6 and 7 are the R code used to simulate combination trials.

### Crossover

**Fig. 6:**
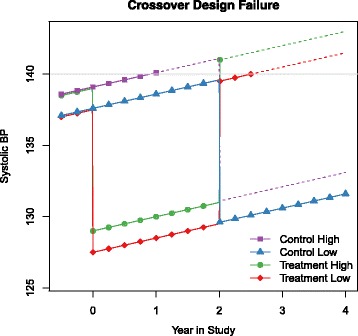
Control high and treatment high have similar starting values as does control low and treatment low. Both treatments are diagnosed as hypertensive while only the control high is diagnosed. This impacts upon our ability to determine if there is a carryover as the treatment group is more likely to be diagnosed irrelevant of the carryover

Figure 6 illustrates why the crossover design fails and why even slight trends over time are problematic. The graph shows noise-free BP patterns for two treatment:control pairs of individuals, one with baseline systolic pressure of approximately 137.5 mm Hg and one with baseline systolic pressure of approximately 139 mm Hg, and with a trend of 1 mm Hg/year. The trend destroys the symmetry of the design: in the pair with higher baseline pressure, the control individual is diagnosed during the first period and is not available in the second period. In the pair with lower baseline pressure both individuals are available for both time periods. The effect of the trend is to selectively remove higher-risk individuals from the control-first arm of the trial.

Figure 6 shows that the difference in cumulative diagnosis without carryover in this example is biased. The dotted lines indicate that the participants have started treatment as systolic hypertension was diagnosed at the last measurement. Of the control pairs only the one with high base line pressure is diagnosed in the first period and not available in the second, in the treatment pairs both are diagnosed in the second period. The effect of the trend is to selectively remove higher-risk individuals from the control-first arm of the trial and results in a bias when testing for carryover.

Figure 5 is consistent with the above. When there is no trend in the data, less error, and measurements are frequent, rates of false positives are close to acceptable which we saw in our simulations in section ‘Crossover design’. Systolic BP with a trend of 0 mm Hg is possible in a short study but not likely over a long period which limits applicability.

## Conclusions

In conclusion, a simple comparison of cumulative incidence in a cross-over provides a valid test for carryover effects only when there is no trend in the underlying outcome variable (or the trial is too short for the trend to be apparent). Unfortunately, BP in prehypertensives and fasting glucose in prediabetics both exhibit non-negligible trends.

Our results have been framed in terms of a discrete intervention period where the treatment effect is constant over time, as might be plausible for a pharmacological intervention. Introducing the additional complications of time-varying effect that would be expected in lifestyle interventions would complicate the analysis but would be unlikely to remove the bias.

These results will depend on the variability in systolic and diastolic BP over time, and on the way in which any carryover effect decreases with time. We have used a simplified model for this which may limit our results.

It does not appear possible to design a parallel-group or cross-over study where carryover effects of this sort can be estimated by simple comparisons of cumulative incidence. Valid tests for carryover effects will require the development of new analytic techniques; the fact that post-diagnosis observations are removed based only on the values of pre-diagnosis observations means that they are Missing At Random and suggests a mixed-model approach.

## Abbreviations

BP, blood pressure; TROPHY, trial of preventing hypertension

## Additional files

Additional file 1 Was used to simulate parallel trials. (R 5 kb)

Additional file 2 Was used to simulate parallel trials where diagnosis is defined using the BP measurements without error. (R 5 kb)

Additional file 3 Was used to simulate parallel trials where the lower bound of the inclusion criteria varied. (R 4 kb)

Additional file 4 Was used to simulate crossover trials. (R 5 kb)

Additional file 5 Was used to simulate crossover trials where diagnosis is defined using the BP measurements without error. (R 5 kb)

Additional file 6 Was used to simulate trials which included both crossover, parallel, and control arms. (R 3 kb)

Additional file 7 Was used to simulate trials which included both crossover, parallel, and control arms when diagnosis is determined by using the BP measurements without error. (R 5 kb)
